# Spatiotemporal metabolic mapping reveals diet-independent remodeling of the postnatal mouse brain

**DOI:** 10.1038/s44324-025-00098-7

**Published:** 2026-02-10

**Authors:** Elisa M. York, Anne Miller, Sylwia A. Stopka, Nathalie Y. R. Agar, Gary Yellen

**Affiliations:** 1 Department of Neurobiology, Harvard Medical School, Boston, MA USA; 2 Molecular Biology, Cellular Biology, and Biochemistry, Brown University, Providence, RI USA; 3 Center for Pathobiochemistry and Genetics, Medical University of Vienna, Vienna, Austria; 4 Department of Neurosurgery, Brigham and Women’s Hospital, Boston, MA USA; 5 Department of Radiology, Brigham and Women’s Hospital, Harvard Medical School, Boston, MA USA; 6 Department of Cancer Biology, Dana-Farber Cancer Institute; Harvard Medical School, Boston, MA USA

**Keywords:** Biochemistry, Biological techniques, Developmental biology, Neuroscience, Physiology

## Abstract

Developing cells undergo extensive metabolic adaptations to support growth and differentiation. Here, using spatially resolved mass spectrometry imaging and stable isotope tracing, we systematically investigate metabolic remodeling in mouse brains at postnatal day 14 and day 28, a period coinciding with the transition from a maternal milk diet to solid food. Untargeted metabolomics reveals global shifts in lipid composition, and region-specific remodeling of central energy metabolism, including increased glycolytic intermediates in grey matter-enriched regions and a global decrease in tricarboxylic acid (TCA) cycle metabolites after weaning. Despite these marked changes in metabolite levels, the glucose incorporation rate remains constant across these developmental stages. Notably, weaning mice onto a milk-replacement diet demonstrates that the observed metabolic adaptations are largely diet-independent. Together, our data suggest that postnatal brain metabolic remodeling is an intrinsically programmed feature of maturation providing region-specific metabolic reorganization to support developmental demands.

## Introduction

All mammals begin life on a maternal milk diet during a critical window of intense brain growth, structural organization, and functional development, before transitioning to solid foods. Unlike many other organs, the mammalian brain is immature at birth and undergoes rapid postnatal development marked by profound changes in neuronal properties alongside proliferation of other cell types, such as astrocytes and oligodendrocytes^1,2^. This intense period of maturation establishes the foundational architecture essential for later cognitive and behavioral function. Metabolic regulation plays a central role during this developmental window, not only by meeting energy demands, but also by supplying biosynthetic precursors needed for cell proliferation, migration, synaptogenesis and myelination^3^. Beyond bioenergetic functions, metabolites and metabolic enzymes themselves can modulate cell signaling events and the epigenetic landscape, thereby influencing neural maturation^4^. Early after birth, ketone bodies serve as major substrates, supporting lipid and amino acid synthesis critical for cell growth and myelination, while glucose utilization only gradually increases over time^5–7^. Genetic models highlight the importance of ketolysis in the neonatal brain, as mice deficient in Succinyl-CoA:3-ketoacid CoA transferase fail to metabolize ketone bodies and show compensatory high glucose consumption, severe hypoglycemia, and early lethality^8^. During that time the components of maternal milk, such as medium-chain fatty acids, phospholipids, myo-inositol, and docosahexaenoic acid, support brain lipid synthesis, thereby shaping synapse formation and cognitive outcomes, while diets high in sugar and saturated fat may impair development^9–13^. The weaning period (at approximately day 21 in mice and 6 months in humans) involves a dietary shift when typically carbohydrate-rich solid food is introduced into the diet. At the same time, the fuel preference of the developing brain also evolves substantially. This transition coincides with a *systemic* rise in glucose concentrations, while circulating β-hydroxybutyrate levels decline during the first weeks after birth^6,14^. Concomitantly, *regional* cerebral glucose metabolism increases, reflecting advancing neuronal activity and maturation, with posterior brain regions exhibiting higher glucose utilization consistent with their developmental timeline^14^.

While prior studies have characterized substrate availability and selected enzyme activities during postnatal brain development, a comprehensive spatial and temporal metabolome “map” across distinct brain regions during early maturation has been lacking. In addition, it remains unclear whether the dietary switch at weaning is a necessary trigger for metabolic brain maturation or if these changes are intrinsically programmed. Here, we employ mass spectrometry imaging to provide spatially and temporally resolved metabolomic profiling of mouse brains at postnatal day 14 and day 28. Our findings reveal that lipid remodeling is a feature consistently observed across brain regions, accompanied by more region-specific shifts in central energy metabolism. This includes increased levels of lower glycolytic intermediates, particularly in grey matter-enriched regions, alongside globally decreased levels of TCA cycle metabolites. Stable isotope tracing with ^13^C₆-glucose in acute hippocampal slices demonstrated similar regional labeling dynamics at both ages suggesting that glucose incorporation relative to pool size is constant. Importantly, these metabolic transitions occur independently of dietary composition, as mice maintained on a milk-replacement diet (mimicking maternal milk) exhibited similar metabolic signatures to littermates weaned onto standard rodent chow. Together, these results suggest that postnatal brain metabolic maturation is largely governed by intrinsic developmental programs rather than the dietary change at weaning.

## Results

### Spatially resolved untargeted metabolomics profiling reveals global metabolic adaptation from postnatal day 14 to day 28

**Fig. 1:**
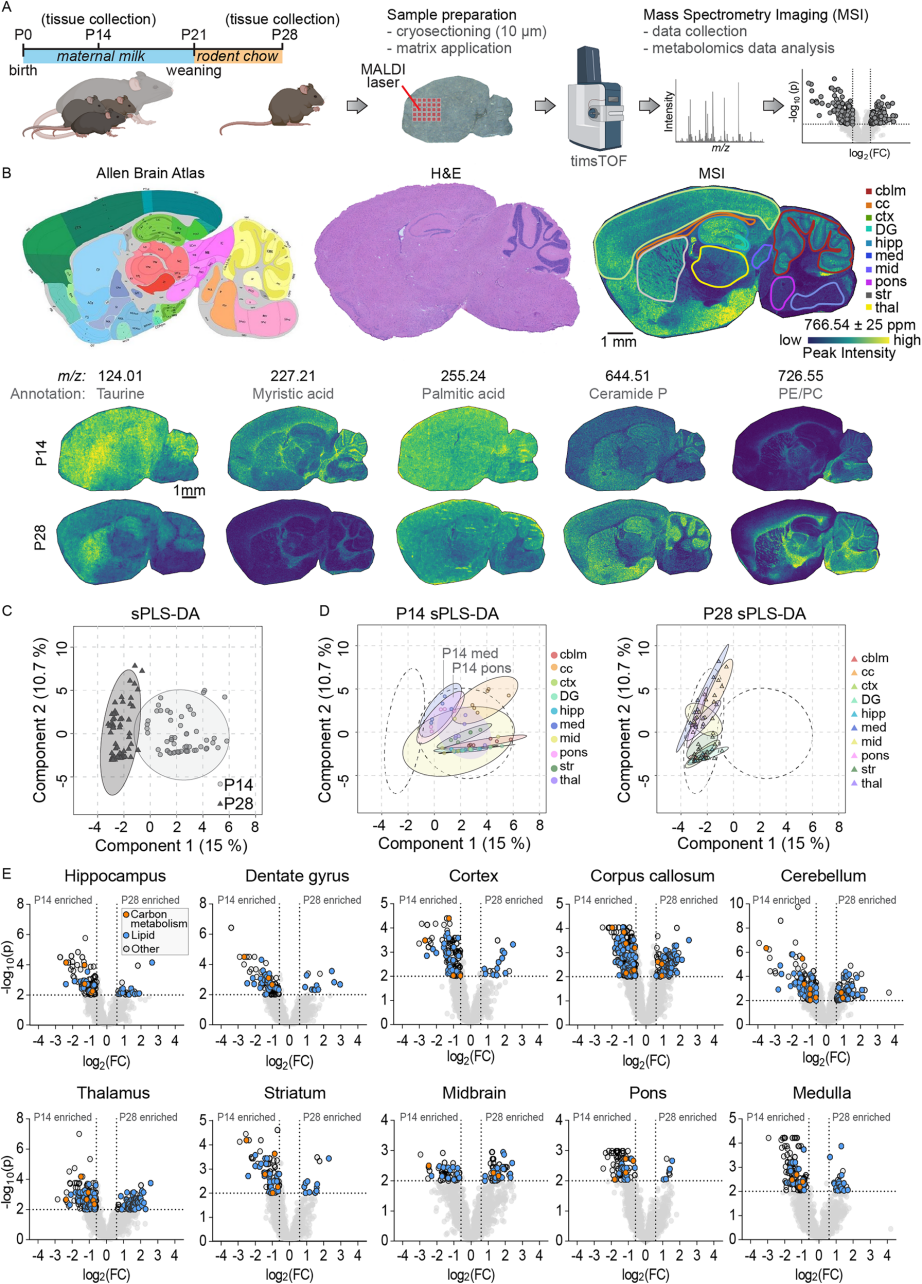
Spatially resolved untargeted metabolomics of mouse brain at postnatal day 14 (P14) and day 28 (P28). **A** Experimental timeline depicting brain collection from P14 pups nursing on maternal milk and from P28 littermates weaned onto standard rodent chow. Samples were sagittally sectioned and prepared for mass spectrometry imaging (MSI) followed by untargeted metabolomics analysis. **B** Indicated regions of interest outlined on the brain sections according to the Allen Brain Atlas (left), H&E stain (middle), and MSI (right), with representative MSI images shown for P14 (top) and P28 (bottom) brains with putative metabolite annotations. **C** Sparse partial least squares discriminant analysis (sPLS-DA) of untargeted metabolomic data from all brain regions, showing separation of P14 (light circles) and P28 (dark triangles) data. **D** sPLS-DA results separated by brain region, illustrating metabolic profiles at P14 (left) and P28 (right). **E** Untargeted metabolomic comparisons between P14 and P28 samples using MetaboAnalyst for each brain region. Peaks with lipid annotations are highlighted in blue, peaks associated with carbon metabolism are shown in orange. cblm cerebellum, cc corpus callosum, ctx cortex, DG dentate gyrus, hipp hippocampus, med medulla, mid midbrain, str striatum, thal thalamus. *N* = 6 P14 (4 female, 2 male) and 6 P28 (4 female, 2 male).

To investigate whether the weaning period is associated with global metabolic changes in the brain, we analyzed sagittal brain sections from mice at postnatal day 14 (one week before weaning from maternal milk) and postnatal day 28 (one week after weaning onto standard chow) (Fig. 1A). Brains were rapidly flash-frozen, cryosectioned at 20 µm thickness, and mounted onto indium tin oxide (ITO)-coated slides for mass spectrometry imaging (MSI). This technique uses a laser to desorb and ionize molecules directly from the tissue, generating mass spectra with spatial resolution, enabling simultaneous mapping of metabolite localization and relative abundance across tissue regions^15,16^. While samples were processed with minimal delay to preserve metabolite integrity, it is important to note that these are postmortem tissues. Transient metabolic activity can persist after death and may affect the abundance of labile metabolites^17^. We then delineated major anatomical regions including cortex, corpus callosum, hippocampus (including dentate gyrus), dentate gyrus only, thalamus, striatum, midbrain, cerebellar cortex, pons, and medulla (Fig. 1B). The resulting ion images not only highlight these different anatomical regions but also revealed striking metabolic differences between the two developmental stages, indicating broad spatial and temporal remodeling of brain metabolism during the weaning transition. Putative annotations indicated decreased taurine (m/z 124.01) and myristic acid (m/z 227.21) from postnatal day 14 to 28, and increased levels of ceramide phosphate (m/z 644.51) and a lipid likely representing phosphatidylethanolamine or phosphatidylcholine (m/z 726.55). An untargeted analysis of these changes between developmental timepoints using MetaboAnalyst revealed a clear separation between postnatal day 14 and day 28 brains by sparse Partial Least Squares Discriminant Analysis (sPLS-DA) (Fig. 1C)^18,19^. When analyzing the individual brain regions separately, samples from postnatal day 14 and day 28 still clustered distinctly, although regional variation was evident (Fig. 1D). Pons and medulla samples from day 14 cluster more toward day 28 samples, potentially reflecting the faster maturation trajectory of these phylogenetically older caudal brain regions^20^. To identify the metabolites contributing to these separations in the sPLS-DA, we generated volcano plots for each region, which revealed numerous mass peaks significantly enriched at either day 14 or day 28 (Fig. 1E). Automated peak annotation putatively assigned many of these as lipids and metabolites related to central energy metabolism. Further unsupervised sub-clustering of the lipid-associated peaks revealed extensive changes in the corpus callosum and thalamus, while only minor changes were observed in the pons, midbrain and medulla between postnatal day 14 and 28 (Fig. S1). Among the most significantly altered lipid classes were sphingolipids, fatty acids, glycerolipids, and glycerophospholipids. Together, these data reveal robust, regionally distinct shifts in both lipid and energy metabolite composition between pre- and post-weaning stages across multiple anatomical brain regions.

### Targeted metabolic analysis reveals region-specific remodeling of core energy and biosynthetic pathways during the weaning transition

**Fig. 2:**
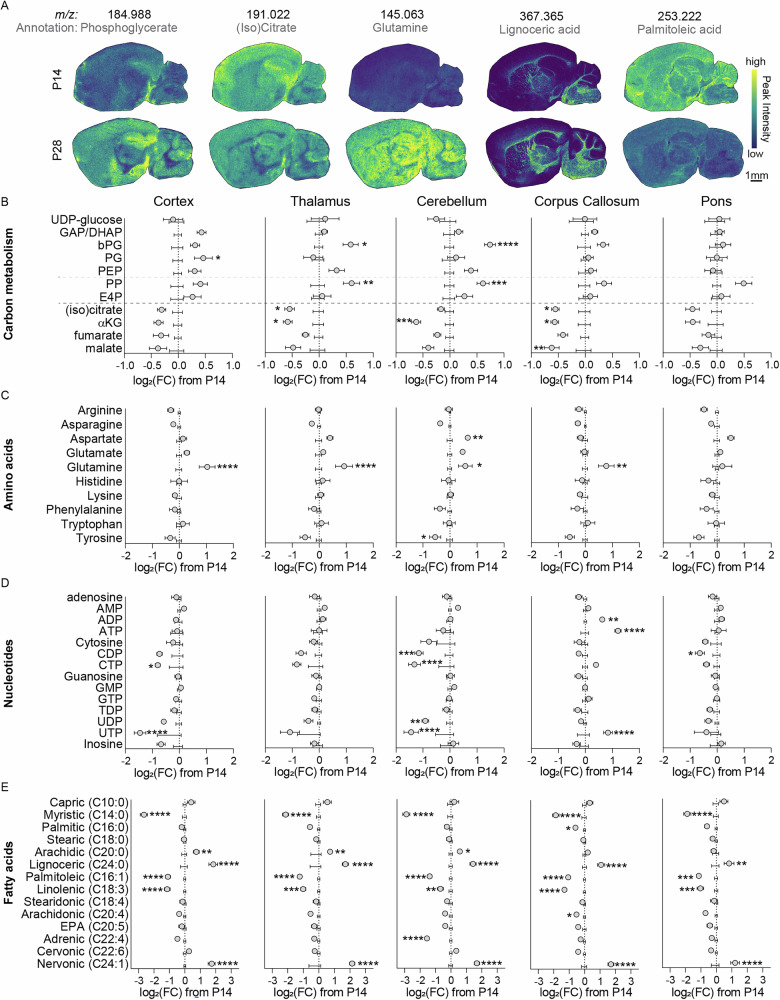
Targeted metabolomic analysis of brain region-specific metabolic profiles from postnatal day 14 (P14) to P28 mouse brain. **A** Representative mass spectrometry images of P14 (top) and P28 (bottom) brain sections showing ion intensities of selected metabolites of interest. **B**-**E** Targeted metabolomic analysis of cortex, thalamus, cerebellum, corpus callosum, and pons. These regions are drawn from the same brains and MSI datasets. Data from P28 brains (grey circles) were plotted relative to P14 ion intensities for metabolites from carbon metabolism (**B**), amino acids (**C**), nucleotides (**D**), and fatty acids (**E**). *p < 0.05, **p < 0.01, ***p < 0.001, ****p < 0.0001 by individual t-tests, with Holm-Šidák multiple comparison correction. *N* = 6 P14 (4 female, 2 male) and 6 P28 (4 female, 2 male). GAP/DHAP glyceraldehyde-3-phosphate/dihydroxyacetone phosphate, bPG bisphosphoglycerate, PG phosphoglycerate, PEP phosphoenolpyruvate, PP pentose phosphates, α-KG α-ketoglutarate.

Given that central energy metabolism was altered in the untargeted analysis, we next investigated the associated pathways in detail using a more targeted analysis. Representative MSI images of selected metabolites are shown in Fig. 2A. Key differences appeared in pathways of central carbon metabolism between postnatal day 14 (center axis) and day 28 (grey circles), including glycolysis, the pentose phosphate pathway, and the TCA cycle (Fig. 2B). A detailed region-specific analysis revealed striking patterns including putatively-assigned glycolytic intermediates glyceraldehyde-3-phosphate/dihydroxyacetone phosphate (GAP/DHAP), bisphosphoglycerate (bPG), phosphoglycerate (PG), and phosphoenolpyruvate (PEP) being increased in cortex, with similar increases for GAP/DHAP and bPG in dentate gyrus and hippocampus, and selective bPG increases in thalamus and cerebellum (Fig. 2B and S2A). In contrast, these metabolites remained largely unchanged or even showed slight decreases in phylogenetically older and white matter-rich regions, such as the corpus callosum, pons, midbrain, and medulla (Fig. 2B and S2A). Among intermediates putatively assigned to the pentose phosphate pathway, we observed robust increases in pentose phosphates across nearly all regions, except corpus callosum and pons where levels remained unchanged, while erythrose-4-phosphate showed more modest or regionally variable increases. On the other hand, many TCA cycle metabolites, including putative (iso)citrate and α-ketoglutarate, decreased in most brain regions with less pronounced reductions in fumarate and malate. The strongest decreases were generally observed in cortex, thalamus, dentate gyrus, hippocampus, and cerebellum, whereas pons showed minimal changes (Fig. 2B and S2A).

To further explore related biosynthetic and catabolic pathways, we also examined putatively assigned amino acids and nucleotides. Among amino acids, glutamine levels increased in cortex, thalamus, cerebellum, corpus callosum, dentate gyrus, hippocampus, and striatum, but remained unchanged in pons, midbrain, and medulla. Aspartate and asparagine were selectively increased in cerebellum and showed mild increases in thalamus and pons, while tyrosine tended to decrease in multiple regions, most notably in cerebellum (Fig. 2C and S2B). For putative nucleotides, CDP, CTP, and UTP levels decreased broadly across brain regions, with the notable exception of the corpus callosum, where CDP and CTP were maintained and UTP was even increased. UDP trended downward in most regions but was unchanged in corpus callosum and pons. Additionally, corpus callosum uniquely displayed increased ADP and ATP levels, whereas these nucleotides were largely stable elsewhere (Fig. 2D and S2C).

Lastly, changes in putative fatty acid composition were broadly consistent across brain regions (Fig. 2E and S2D). We observed decreases in myristic acid (14:0), palmitoleic acid (16:1), and linolenic acid (18:3) in all regions, as well as regional decreases in arachidonic acid (20:4, corpus callosum and medulla) and adrenic acid (22:4, cerebellum). In contrast, increases were detected in arachidic acid (20:0, cortex, thalamus, cerebellum, dentate gyrus, hippocampus, and midbrain), lignoceric acid (24:0, cortex, thalamus, cerebellum, corpus callosum, pons, hippocampus, striatum, with a trend in medulla), and nervonic acid (24:1, cortex, thalamus, cerebellum, corpus callosum, pons, hippocampus, striatum, midbrain, and medulla).

Together, these results highlight distinct region-specific metabolic signatures during the transition from postnatal day 14 to 28, including selective increases in glycolytic intermediates and pentose phosphates, global decreases in TCA cycle metabolites, differential changes in amino acid and nucleotide pools, and a widespread remodeling of fatty acid composition.

### Region-specific glucose tracing reveals preserved pathway flux during postnatal brain maturation

**Fig. 3:**
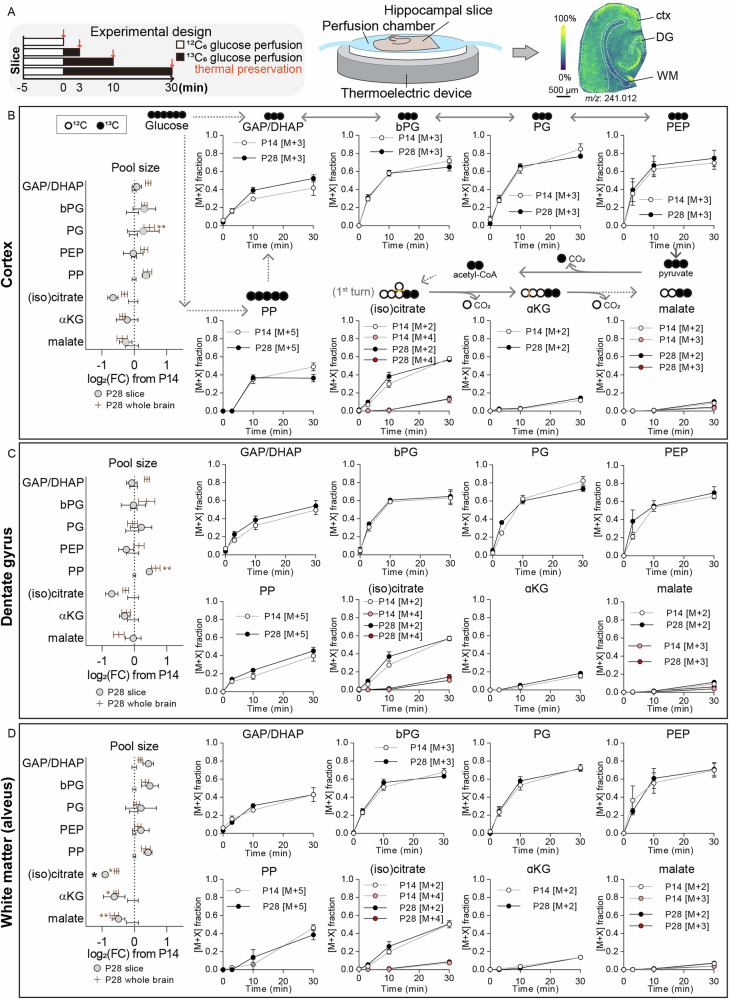
Region-specific glucose tracing reveals preserved pathway activity during postnatal brain maturation. **A** Schematic of experimental setup: Acute hippocampal slices from postnatal day 14 or 28 mice were perfused for 0, 3, 10, or 30 minutes with artificial cerebrospinal fluid (aCSF) containing ^13^C_6_-glucose (black bars), followed by rapid thermal preservation and mass spectrometry imaging. **B** Labeling dynamics in the cortex. Metabolite labeling diagrams are shown above each graph, depicting ^12^C (open circles) and ^13^C (filled circles). Left panel compares relative metabolite pool sizes between P14 and P28 from slice data (grey circles) and previously shown data from flash-frozen whole brain (brown bars). Right panel plots isotopologue fractions [M + X] over time for P14 (white circles) and P28 (black circles) slices. Corresponding metabolite pool sizes and labeling dynamics are calculated for the dentate gyrus (**C**), and alveus (**D**). *p < 0.05, **p < 0.01 by individual t-tests, with Holm-Šidák multiple comparison correction. *N* = 6 P14 (4 female, 2 male) and 6 P28 (3 female, 3 male). GAP/DHAP glyceraldehyde-3-phosphate/dihydroxyacetone phosphate, bPG bisphosphoglycerate, PG phosphoglycerate, PEP phosphoenolpyruvate, PP pentose phosphates, α-KG α-ketoglutarate.

To investigate metabolic flux rates rather than relative steady-state metabolite levels, we next performed stable isotope tracing in acute hippocampal brain slices. 450-µm-thick acute brain slices containing the entorhinal cortex and hippocampus were submerged in a physiological chamber with constant flow of oxygenated artificial cerebrospinal fluid in the absence or presence of uniformly labeled ^13^C_6_-glucose, allowing us to track downstream ^13^C incorporation in different hippocampal regions (cortex, dentate gyrus, and white matter/alveus) in mice aged postnatal day 14 and 28 (Fig. 3A). We first verified that the metabolite changes we previously observed in the brain at these ages are mirrored in the slices, confirming that the slice system accurately reflects in vivo metabolic states. As expected, the ex vivo slices recapitulated the observed increases in glycolytic intermediates and decreases in TCA cycle intermediates from day 14 to day 28 (Fig. 3B–D**, left**). We then analyzed the main isotopologues detected only in ^13^C_6_ glucose-treated tissue, but not in the unlabeled controls, allowing us to assess its incorporation into glycolysis, the pentose phosphate pathway, and the TCA cycle. Glycolytic intermediates approached a (pseudo) steady-state labeling within 30 minutes, with approximately 40-70% average ^13^C incorporation across all analyzed regions (somewhat consistent with previous NMR studies, showing maximal ^13^C-glucose incorporation into TCA cycle intermediates already after around 15 minutes^21^). Pentose phosphate intermediates reached a steady-state labeling level within 10 minutes in the cortex (approximately 40% labeling efficiency), but labeling was delayed ( > 30 minutes) in the dentate gyrus and alveus (Fig. 3B–D). TCA cycle intermediates were predominantly labeled in the first round (meaning that M + 2 isotopologues were predominant), with (iso)citrate showing the highest labeling (50-60% in all regions), whereas α-ketoglutarate and malate showed lower labeling (approximately 10-20% in all regions, with malate labeling below 5% in white matter (Fig. 3B–D). Importantly, labeling dynamics did not differ between slices from 14- and 28-day-old mice, indicating that although metabolite levels change, the flux relative to pool size is constant. This suggests that pathway activity is scaled proportionally rather than fundamentally reprogrammed.

### Brain metabolic remodeling during postnatal development is evident in gene expression profiles and remains unaltered by a milk-replacement diet

**Fig. 4:**
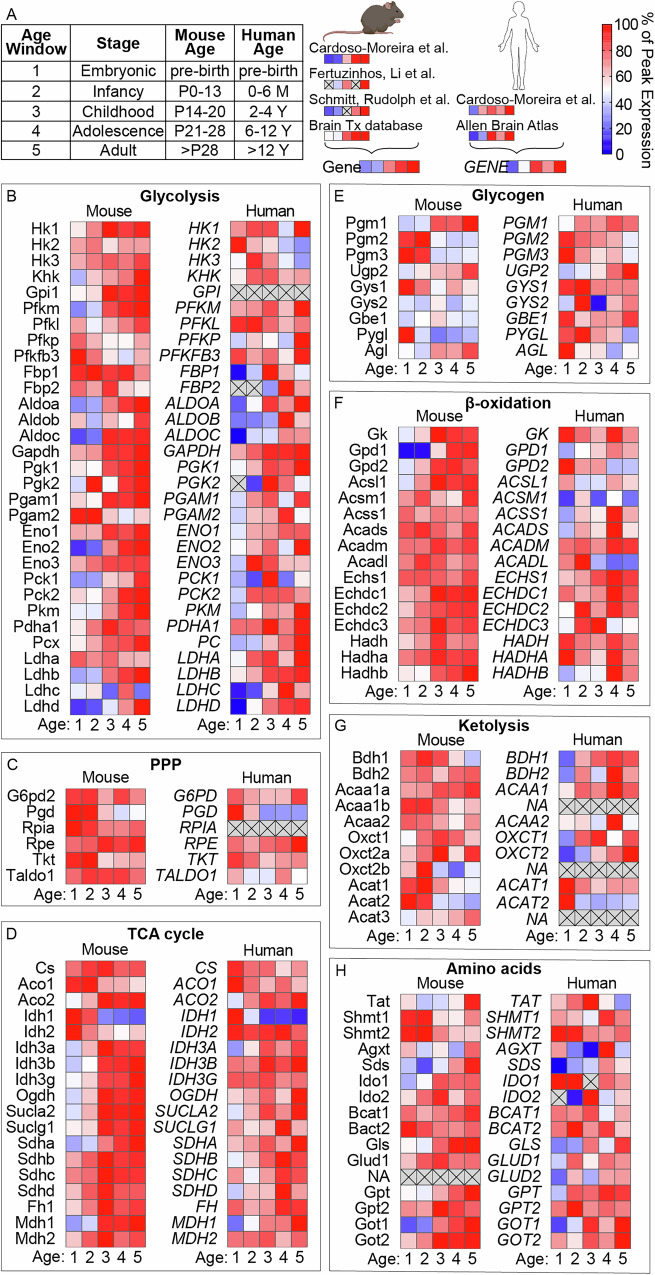
Gene expression profiles reveal brain metabolic remodeling during postnatal development. **A** Gene expression data were compiled from four independent transcriptomic datasets for mouse brain and two for human brain. To enable comparison across datasets, samples were grouped into developmental age windows and expression levels were normalized to the peak of expression of each gene within its respective dataset. Normalized gene expression profiles for key metabolic pathways across postnatal development: **B** glycolysis, **C** pentose phosphate pathway (PPP), **D** tricarboxylic acid (TCA) cycle, **E** glycogen metabolism, **F** β-oxidation, **G** ketone body metabolism (ketolysis), and **H** amino acid metabolism.

**Fig. 5:**
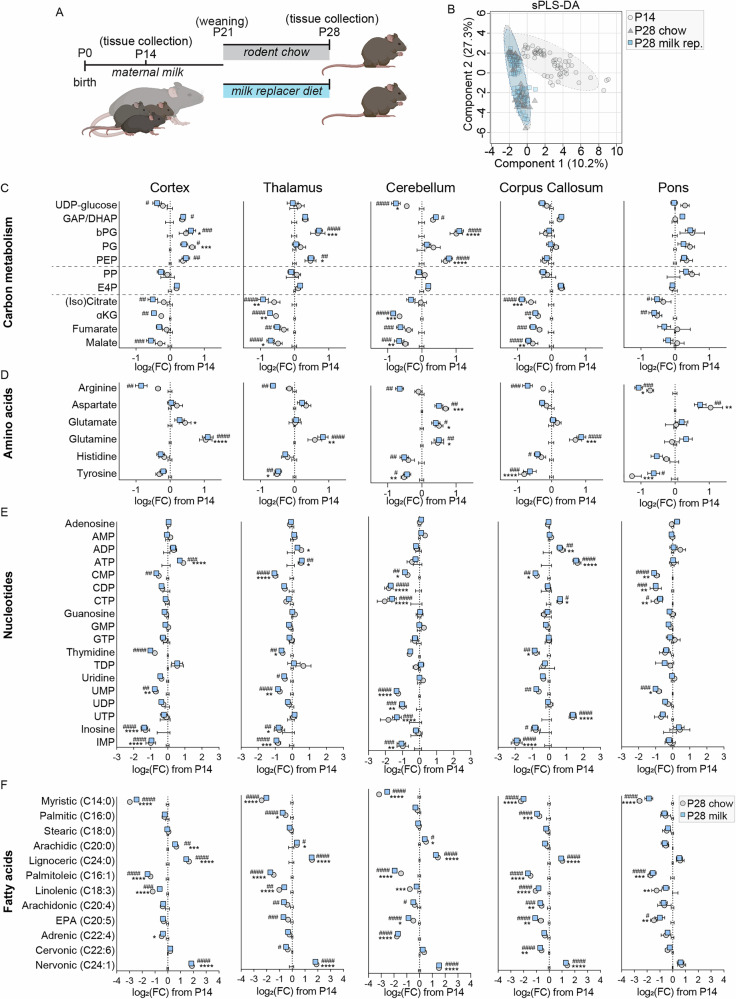
Brain metabolic profiles assessed under standard chow and milk-replacement diets. **A** Experimental design: Brains were collected at postnatal day 14 (P14) from pups nursing on maternal milk. Remaining littermates were weaned at P21 onto either standard chow or milk replacer diet and brains were collected at P28. **B** Untargeted metabolomic comparisons between P14 (light grey circles), P28 standard chow (dark grey triangles), or P28 milk replacer diet (blue squares) were visualized using sparse partial least squares discriminant analysis in MetaboAnalyst. **C**-**F** Targeted metabolomic analysis of the cortex, thalamus, cerebellum, corpus callosum, and pons. P28 standard chow (grey circles) and P28 milk replacer diet (blue squares) are plotted relative to P14 values for metabolites related to carbon metabolism (**C**), amino acids (**D**), nucleotides (**E**), and fatty acids (**F**). *p < 0.05, **p < 0.01, ***p < 0.001, ****p < 0.0001 by two-way ANOVA with Tukey multiple comparison correction. *N* = 7 P14 (4 female, 3 male), 7 P28 chow (3 female, 4 male) and 9 P28 milk replacer diet (6 female, 3 male); conducted as 3 separate experiments. GAP/DHAP glyceraldehyde-3-phosphate/dihydroxyacetone phosphate, bPG bisphosphoglycerate, PG phosphoglycerate, PEP phosphoenolpyruvate, PP pentose phosphates, α-KG α-ketoglutarate.

To explore whether our observed metabolic shifts are reflected at the transcriptional level, we analyzed publicly available mouse and human brain gene expression datasets. For comparison between datasets, we grouped samples into developmental windows: embryonic (mouse and human pre-birth), infancy (mouse postnatal day 0–13, human 0–6 months), childhood (mouse day 14–20, human 2–4 years), adolescence (mouse day 21–28, human around 5–12 years), and adult (mouse >28 days, human >12 years) and display data as a percentage of the maximum expression in that dataset (Fig. 4A)^22–26^. Although these data lack regional resolution, they revealed coordinated metabolic adaptations in both mice and humans. Specifically, glycolytic enzyme expression increased with age (Fig. 4B), while pentose phosphate pathway genes showed a slight decrease (Fig. 4C). Most TCA cycle genes either remained stable or increased, except for isocitrate dehydrogenase 1, which decreased in both species (Fig. 4D). Genes related to glycogen metabolism, β-oxidation, and ketolysis showed more heterogeneous regulation patterns (Fig. 4E–G). Finally, amino acid metabolism genes generally increased in mice and displayed more variable regulation in humans, though most trended upward (Fig. 4H). Collectively, these patterns suggest that transcriptional reprogramming of metabolic enzymes is tightly coordinated but not always directly proportional within or across pathways, highlighting the complex regulation of brain maturation at multiple levels of molecular organization. This prompted us to test whether our observed metabolic changes are driven by *intrinsic* developmental programs or shaped by *extrinsic* dietary cues during the weaning event. We therefore placed mice on a milk-replacement diet mimicking maternal milk composition. On postnatal day 21, mice were weaned either onto standard chow (62% carbohydrate, 25% protein, 13% fat) or onto a milk-replacement diet (77% fat, 18% protein, 5% carbohydrate; Figs. 5A and S3A). Body weight gain, tail length, and food intake were monitored, with no significant differences observed between the two groups, indicating comparable overall growth during the dietary intervention period (Fig. S3B-E). MSI analysis of brains collected at day 28 revealed that global metabolomic profiles of mice on the milk-replacement diet closely resembled those of chow-fed 28-day old mice littermates and were clearly distinct from 14-day old brains (Fig. 5B). Specifically, the characteristic increases in glycolytic intermediates, decreases in TCA cycle metabolites, and regional changes in amino acids and nucleotides we had previously observed (Fig. 2), were consistently observed regardless of diet (Figs. 5C-F and S4). Thereby, our quantitative analysis across different brain regions confirmed that the metabolic remodeling from day 21 to day 28 occurred independently of dietary macronutrient composition. Together, these findings demonstrate that postnatal brain metabolic maturation is robust and largely diet-independent, with similar trends evident already at the gene expression level.

## Discussion

In this study, we set out to systematically map how brain metabolism remodels during the postnatal weaning transition in mice. Using spatially resolved metabolomics and stable isotope tracing, we uncover global shifts in metabolite levels across central carbon metabolism, lipid classes, amino acids, and nucleotides. Surprisingly, a dietary intervention with a milk-replacement diet did not alter these metabolic profiles, and glucose tracing revealed stable glucose-derived carbon flow across brain regions and ages, suggesting that pathway activity is scaled proportionally with metabolite pool size.

A general consideration for MSI-based measurements is that analyses are performed on postmortem tissue, where residual enzymatic activity may persist transiently after death. Such processes can alter the abundance of labile metabolites, particularly high-energy intermediates such as ATP or phosphocreatine^17^. While our protocol minimized postmortem delay, we cannot fully exclude those differences in metabolic stability between developmental stages could influence measured pool sizes for all metabolites. Importantly, this limitation is intrinsic to MSI workflows and not specific to the present study. Nevertheless, the consistency between metabolite pool distributions observed in flash-frozen whole-brain tissue and acutely prepared hippocampal slices subjected to rapid heat denaturation strongly supports the robustness of our data. Together, these results validate the reliability of our workflow for comparative developmental metabolomics.

By assessing region-specific changes in putatively-assigned metabolite levels, we identify distinct metabolic profiles across brain regions during this critical postnatal window. The cortex and hippocampus display pronounced accumulation of glycolytic intermediates and increased glutamine levels. In contrast, the corpus callosum shows minimal glycolytic shifts but increased ATP and preserved pyrimidine nucleotides. Regions such as the thalamus and cerebellum exhibit selective increases in bPG and amino acids (aspartate and asparagine). Meanwhile, phylogenetically older regions (pons, midbrain, medulla), show relatively stable metabolic and lipid profiles, potentially indicating an earlier completion of developmental remodeling. Overall, our data suggest that during postnatal maturation, brain regions can adopt defined metabolic programs to support their specific developmental trajectories. The general accumulation of glycolytic intermediates without corresponding increases in TCA cycle metabolites could point to a Warburg-like shift, where glycolysis is redirected toward anabolic processes rather than full oxidative metabolism. Increased pentose phosphate pathway intermediates and glutamine could further support biosynthetic needs, including nucleotide and lipid synthesis required for membrane expansion and synaptic remodeling. Some of these findings align well with previous work, even if the exact time points and organisms are different. Generally, increasing glutamine concentrations together with decreases in taurine could potentially reflect enhanced astrocytic and neuronal metabolic coupling^1,27^. Increases in glycolytic intermediates and pentose phosphate pathway metabolites have been linked to biosynthetic and redox demands, and local cerebral glucose utilization is reported to increase with age in most brain regions, while more posterior regions mature earlier, highlighting region-specific metabolic programming during development^6^. Interestingly, we also observe an increase in nervonic and lignoceric acid, as previously reported and suggested to be involved in myelination^28,29^. Recent studies using carbon isotope tracing demonstrated that brain palmitic acid and other fatty acids are largely maintained through local de novo lipogenesis from dietary sugars, rather than direct dietary uptake^30,31^. Collectively, these observations support the view that the developing brain undergoes region-specific metabolic programming to meet the biosynthetic and energetic requirements of maturation.

While the changes in metabolite pool sizes reflect the steady-state concentrations determined by the balance of production and consumption, *metabolic flux* refers to the rate at which molecules move through their respective pathways. Increased metabolite levels do not necessarily imply increased pathway activity, and they may instead reflect slower consumption, altered storage, or local preparation for different biosynthetic demands. Even though our data showed distinct changes in intermediate pools across several brain regions during maturation, ^13^C-glucose tracing experiments demonstrated that the rate of glucose-derived carbon incorporation into downstream metabolism remained stable between 14-day old vs 28-day old mice. Interestingly, similar effects have been reported in aging, where substantial changes in metabolite pool sizes are seen across tissues, yet fluxes of major circulating metabolites remain stable, suggesting that during physiological transitions like aging or development, metabolic remodeling often involves adjustments in pool sizes and pathway branching rather than flux rates^32^. There are several scenarios that could explain this observation, such as a balanced production and consumption rate, that simply adapted alongside the increased pool size. Alternatively, cells might accumulate glycolytic intermediates to support biosynthesis, or some intermediates, such as those of the TCA cycle, could potentially be “diverted” to anabolic processes such as neurotransmitter or lipid synthesis rather than energy production. Another possibility is the presence of distinct metabolic sub-pools, e.g. one that is quickly exchangeable and one that is not, which could account for our observed labeling plateaus at 30 minutes below 100%.

Lastly, some of our metabolomics data are consistent with the collected available gene expression data, which lack regional specificity. Whereas some general trends overlap with our spatial metabolomics data, some adaptations are region-specific and are not reflected in the transcriptional analysis. This is to be expected, as gene expression does not necessarily reflect enzymatic capacity or correlate with actual flux or metabolite pool sizes. Alternatively, these can be shaped by post-transcriptional regulation, enzyme activity, substrate availability, and local metabolic demands^33^. In the future, additional approaches such as spatial transcriptomics and proteomics could help us gain a more mechanistic understanding of regional metabolic pathway regulation across different layers of molecular organization.

On a technical note, while interpreting MSI data, it is important to consider that physiochemical differences between brain regions could result in varying ionization efficiencies or so-called matrix effects^34,35^. To account for this, we primarily compare between the same brain region in different settings, rather than between different brain regions. Physiologically, remodeling events between day 14 and day 28 likely reflect the complex demands of postnatal brain development, including rapid myelination, synapse formation, and glial expansion^1,36–38^. While the observed changes in lipid composition could partially represent increased myelination, if these shifts were solely driven by myelin synthesis, we would expect major differences in white matter-rich regions such as the corpus callosum, with minimal changes in neuronally enriched regions like the dentate gyrus. Instead, we observed changes across all regions, suggesting additional roles for lipid remodeling in neuronal membranes, organelle composition, and potentially lipid-derived signaling pathways.

Most strikingly, our observed metabolic shifts were maintained regardless of diet, suggesting they are primarily driven by intrinsic developmental programs rather than by changes in macronutrient composition. Interestingly, IDH1 was the only TCA-related gene found to be downregulated in both mouse and human datasets. IDH1 is primarily localized to the cytosol, where it produces α-ketoglutarate and NADPH, linking it to redox regulation, anaplerosis, and epigenetic cofactor supply^39^. In the context of reduced TCA metabolite levels, this may reflect a decrease in cytosolic anaplerotic input, or alternatively, reduced α-ketoglutarate availability could influence developmentally regulated epigenetic modifications, such as histone and DNA demethylation. Together with the gene expression data, this might suggest that transcriptional programs set the stage for metabolic remodeling, although final steady-state pool sizes may be additionally shaped by downstream usage and compartmentalization. The triggers for this developmental metabolic reprogramming remain unclear. In vivo ^13^C-glucose infusion at postnatal day 14 and day 28 would further aid in distinguishing whether observed changes reflect substrate preference or pool remodeling. Our dietary intervention focused on mimicking the fat-to-carbohydrate ratio of maternal milk, but specific lipid species, micronutrients, or bioactive compounds unique to milk may still influence metabolism. Additionally, other systemic signals, such as the permeability of the blood-brain-barrier, hormonal changes, neuronal activity patterns, or circulating growth factors, could drive these shifts^9,14,40,41^. Finally, we did not investigate the role of the microbiome in shaping nutrient availability or metabolic signaling to the brain. Microbial metabolites might indirectly influence brain metabolism and warrant future exploration^42,43^.

In summary, we demonstrate that postnatal brain maturation involves robust, region-specific remodeling of metabolite pools, notably increased glycolytic intermediates and decreased TCA metabolites without a major change in glucose-derived flux and independent of diet macronutrient composition. Together, these findings highlight a developmentally encoded metabolic program that likely supports biosynthetic and functional demands during brain maturation, rather than simply reflecting dietary adaptation.

## Methods

### Mice and dietary intervention

Both male and female wild-type C57BL/6 N mice at postnatal day (P)14 and P28 were included in all experiments; however, the study was not powered to detect sex-specific effects, and data from both sexes were pooled for analysis. Animals were bred in-house and housed in ventilated cages within a barrier facility maintained on a 12/12 h light/dark cycle with controlled temperature (24 °C) and humidity (53%). Food and water were provided ad libitum. Dams were fed Picolab standard chow (Rodent Diet 5053). At P21, offspring were weaned and randomly assigned to either remain on standard chow or switch to a milk-replacer diet (Research Diets, Inc., L16040901), formulated to mimic the macronutrient composition of maternal milk. Littermates were balanced across groups with respect to sex wherever possible. Mice had continuous access to their assigned diet: chow was provided in wire-top hoppers, while the milk-replacer, a soft paste, was placed in a food tray on the cage floor. To monitor intake, fresh food was weighed daily before presentation, and any remaining food (including spillage) was collected and reweighed after 24 hours. All mice were weighed daily to assess growth trajectories. Tail lengths were measured and recorded as a proxy of mouse growth and size. All experiments were conducted in accordance with the NIH Guide for the Care and Use of Laboratory Animals and the Animal Welfare Act. Protocols were approved by the Harvard Medical Area Standing Committee on Animals (institutional animal welfare assurance no. D16-00270 (A3431-01), protocol no. IS00001113-6).

### Brain collection, acute hippocampal slice preparation, perfusion and stable isotope tracing

At postnatal day 14 or 28, mice were weighed, deeply anesthetized with isoflurane, and decapitated. The brain was extracted within 15 seconds and immediately transferred to a pre-cooled cryovial positioned in liquid nitrogen. The total time from decapitation to complete freezing did not exceed 30 seconds to minimize post-mortem metabolic degradation. Direct immersion of the cryovial into liquid nitrogen ensured rapid vitrification; no intermediate steps (such as cooling on ice) were performed. Samples were then kept at −80 °C until further processing.

The acute hippocampal slice experiments were performed as previously described^44,45^. On the day of each experiment, a mouse was anesthetized with isoflurane and decapitated. The brain was rapidly extracted, and the cerebellum and prefrontal cortex anterior to the optic chiasm were removed. The remaining tissue, including the hippocampus, was submerged in ice-cold slicing solution containing: 87 mM NaCl, 2.5 mM KCl, 1.25 mM NaH₂PO₄, 25 mM NaHCO₃, 7 mM MgCl₂, 0.5 mM CaCl₂, 25 mM d-glucose, and 75 mM sucrose (osmolality ~310 mmol kg–1, pH ~7.4). The tissue was glued by its dorsal surface to the stage of a vibrating Compresstome (Precisionary Instruments) and embedded in 2% agarose. Transverse hippocampal slices (450 µm thick) were cut into cold slicing solution and transferred using a glass pipette into 37 °C artificial cerebrospinal fluid (aCSF) containing: 120 mM NaCl, 2.5 mM KCl, 1 mM NaH₂PO₄, 26 mM NaHCO₃, 1 mM MgCl₂, 2 mM CaCl₂, and 10 mM d-glucose ( ~ 300 mmol kg–1, pH 7.4). The osmolarity of all solutions was checked frequently (Wescor, VAPRO 5520). Slices were recovered for a minimum of 30 minutes in oxygenated aCSF at 34°C before performing experiments. We noticed no differences in metabolite levels from slices that were recovered between 30 min - 3 hours^46–48^. All solutions were continuously bubbled with 95% O₂ and 5% CO₂. For physiological experiments, slices were placed in a chamber with a thermoelectric bottom to enable fast thermal preservation as previously published^45^. In ^13^C₆-glucose tracing experiments, glucose in aCSF was replaced by 10 mM ^13^C₆-glucose (Cambridge Isotope Laboratories, CLM-1396) and perfused for3, 10 or 30 minutes. Control slices without label were included in each experiment.

### Sample preparation for MSI

Flash-frozen brains and slices were cut at 10 μm thickness (Microm HM 550, Thermo Scientific) and thaw-mounted onto ITO slides (MALDI IntelliSlides, Bruker Daltonics). Slides were scanned (Epson Perfection 4490 Photo, 2400 dpi) for optical images and desiccated before matrix application. To prepare the matrix, 39.5 mg 1,5-diaminonaphthalene (DAN) was dissolved in 500 μl 1 M HCl and 4 ml H₂O, vortexed, sonicated, and mixed with 4.5 ml ethanol and 100 µM ^15^N_1_-glutamate ([M-H], 147.0418), 1 µM ^15^N_5_-AMP ([M-H], 351.044) and 10 µM ^15^N_5_-ATP ([M-H], 510.9692) were added to the matrix. The matrix was then loaded into an HTX TM-Sprayer (HTX Technologies) and applied at 0.09 ml/min, nozzle velocity 1,200 mm/min, 75 °C, 10 psi, with four passes at 2 mm spacing.

Mass spectra were acquired on a timsTOF fleX (Bruker Daltonics) in negative-ion mode at 20 µm resolution. Most metabolites were acquired in one run (m/z 50–1,000), with a second method targeting small molecules (m/z 50–400). Each MSI pixel included 1,000 laser shots at 10 kHz. Instrument parameters were optimized according to mass range and analyte selectivity. Calibration was performed using the spiked ^15^N-labelled standards. For the small-molecule method, a calibration spot with lactate, pyruvate, and ^15^N₁-glutamate was added before matrix application.

### MALDI imaging data analysis and natural abundance isotope correction

Data were analyzed as previously published^44,45^. Raw data files were analyzed in SCiLS Lab (Bruker Daltonics). Total ion current normalization was applied, and spectra were exported as CSV, peak-picked (generally 0.05% relative intensity), and processed in mMass 5.5.0. Peak annotations were matched to an in-house library and exported for further analysis. For our targeted analyses, metabolites were verified by MS/MS and ion mobility with respect to standards (previously published in Miller et al., 2023^45^. For stable isotope tracing, control slices without label were used to exclude non-specific signals. Natural abundance correction was performed using a matrix-based method as previously published^49^. Data are plotted as the abundance of each independent isotopologue (M + X), divided by the total sum of all isotopologues to give a labeling fraction.

### Untargeted data analysis with MetaboAnalyst and peak annotations

Exported peak lists from each sample and brain region were exported from mMass and uploaded to the MetaboAnalyst server (https://www.metaboanalyst.ca/) as postnatal day 14 or 28 groups, either with or without regional binning. A one-factor statistical analysis was performed with a 0.01 mass tolerance and no data normalization. To account for multiple testing, false discovery rate (FDR) correction with a threshold of 0.05 was enabled in the generation of volcano plots, which were produced with a fold change limit of 1.5 and a *p*-value cutoff of 0.01. *P*-value distributions were visually inspected to ensure proper correction and data quality. For supervised classification, sparse partial least squares discriminant analysis (sPLS-DA) was performed using the MetaboAnalyst implementation, which applies cross-validation to determine the optimal number of components and prevent model overfitting. Feature selection within sPLS-DA was guided by variable importance in projection (VIP) scores, and model validation followed the procedures described by Xia et al.^50^. To assign annotations to detected metabolites in an unsupervised manner, we used a multi-step classification pipeline. First, compound names from experimental datasets were classified into functional categories using a supervised text classification approach. A Naive Bayes classifier was trained on a manually curated dataset of known metabolites^51^. This classifier was trained to distinguish between major classes such as lipids, amino acids/peptides, purines/pyrimidines, carbon metabolism, and others. Then, for compounds predicted as lipids, subclass information was extracted using the Goslin lipid parser^52^. This tool parses systematic lipid names to identify the lipid headgroup and compute total side chain carbon counts and double bond numbers. The annotated compound dataset was then merged with differential abundance data using the measured m/z value as a matching key. The combined dataset included both biochemical annotations and statistical significance metrics and are available in the provided source data file.

### Transcriptomic data assembly

Publicly available developmental transcriptomic datasets^22–26^ were mined for metabolic enzymes involved in energy metabolism. To facilitate comparison across experimental protocols, enzyme expression was calculated as a percentage of its maximum expression within each data set. Timepoints of expression were binned into 5 age windows: embryonic (mouse and human pre-birth), infancy (mouse postnatal day 0–13, human 0–6 months), childhood (mouse day 14–20, human 2–4 years), adolescence (mouse day 21–28, human around 5–12 years), and adult (mouse >28 days, human >12 years). The average relative expression value for each enzyme in each time window was then calculated and plotted as a heatmap, allowing visualization of data from several transcriptomic resources. Relative enzyme expression levels could then be compared for several metabolic pathways, across time and species.

### Quantification and statistical analysis

Specific statistical details are provided in figure legends. Data are presented as mean ± s.e.m., and biological sample size for each experiment are indicated. These sample sizes were chosen based on an initial power analysis performed with pilot data (alpha value: 0.05; power value: 0.8; effect size: 2). No data sets were excluded. Metabolite ion intensities were normalized to either the P14 data (whole brain experiments) or the untreated control slice (isotope labeling), and are presented as a log_2_ fold change or relative labeled fraction, respectively. Hypothesis testing was performed using two-tailed, paired Student’s t-tests with multiple comparisons correction or two-way ANOVA, as indicated (GraphPad Prism, version 7). Significance levels: ***P < 0.001, **P < 0.01, *P < 0.05.

## Supplementary information

SupplementaryInformation.

## Data Availability

All results are available through our submitted Source Data table.
