# Supplementary material for: Spatiotemporal metabolic mapping reveals diet-independent remodeling of the postnatal mouse brain
Source: NPJ Metab Health Dis. 2026 Feb 10;4:7. doi: 10.1038/s44324-025-00098-7 (PMC12891541; doi:10.1038/s44324-025-00098-7)
Supplement: Supplementary file 1 — SupplementaryInformation. [file 44324_2025_98_MOESM1_ESM.pdf]

Fig S1

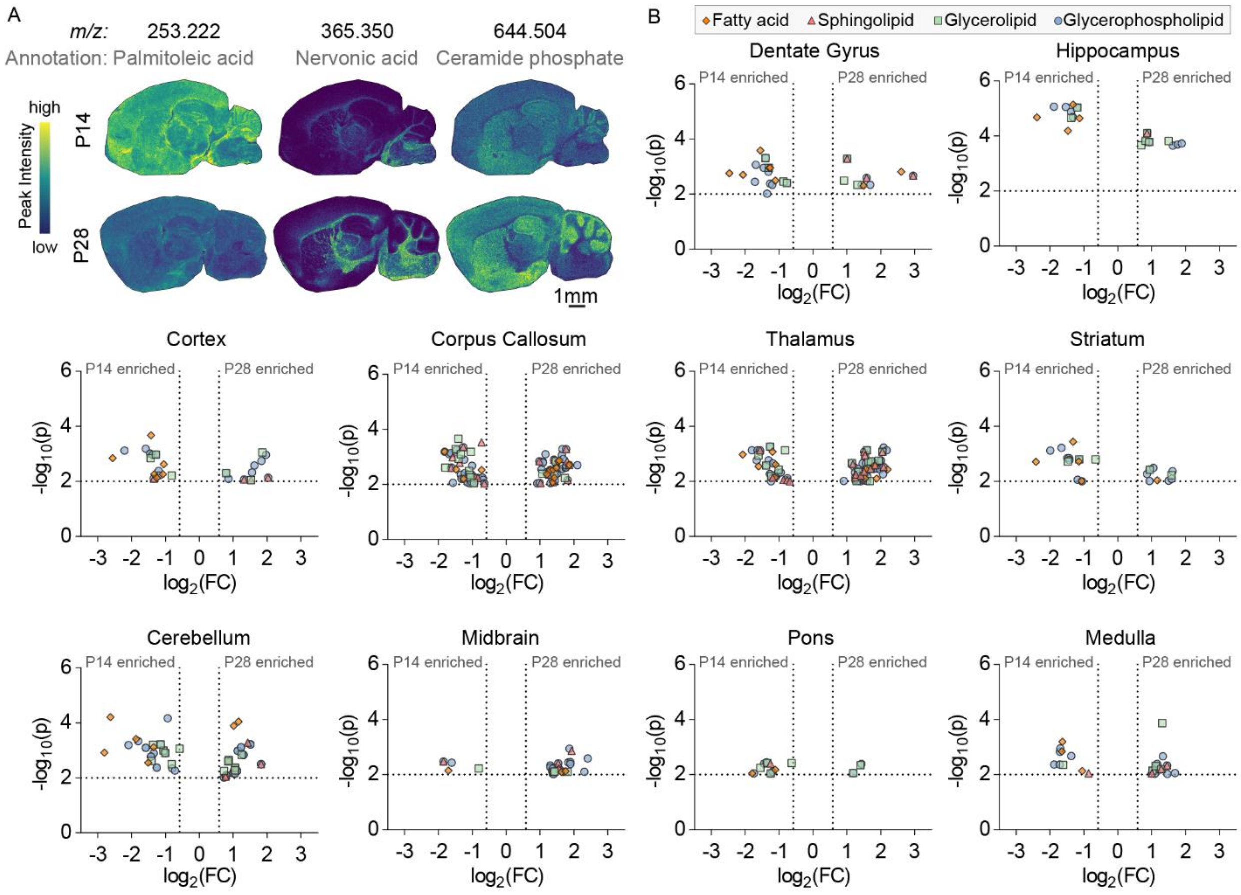

Fig S1 (associated with Fig 1): Untargeted analysis of lipid subclasses. A) Representative ion images of mouse brains at postnatal day 14 (P14; top) and P28 (bottom). B) Volcano plot analysis identified lipid-associated mass features differing between P14 and P28 brains across all regions of interest. These features were classified into lipid subclasses, including fatty acids (orange diamonds), sphingolipids (pink triangles), glycerolipids (green squares), and glycerophospholipids (blue circles) using a lipid subclass parser based on compound structural and name features.

Fig S2

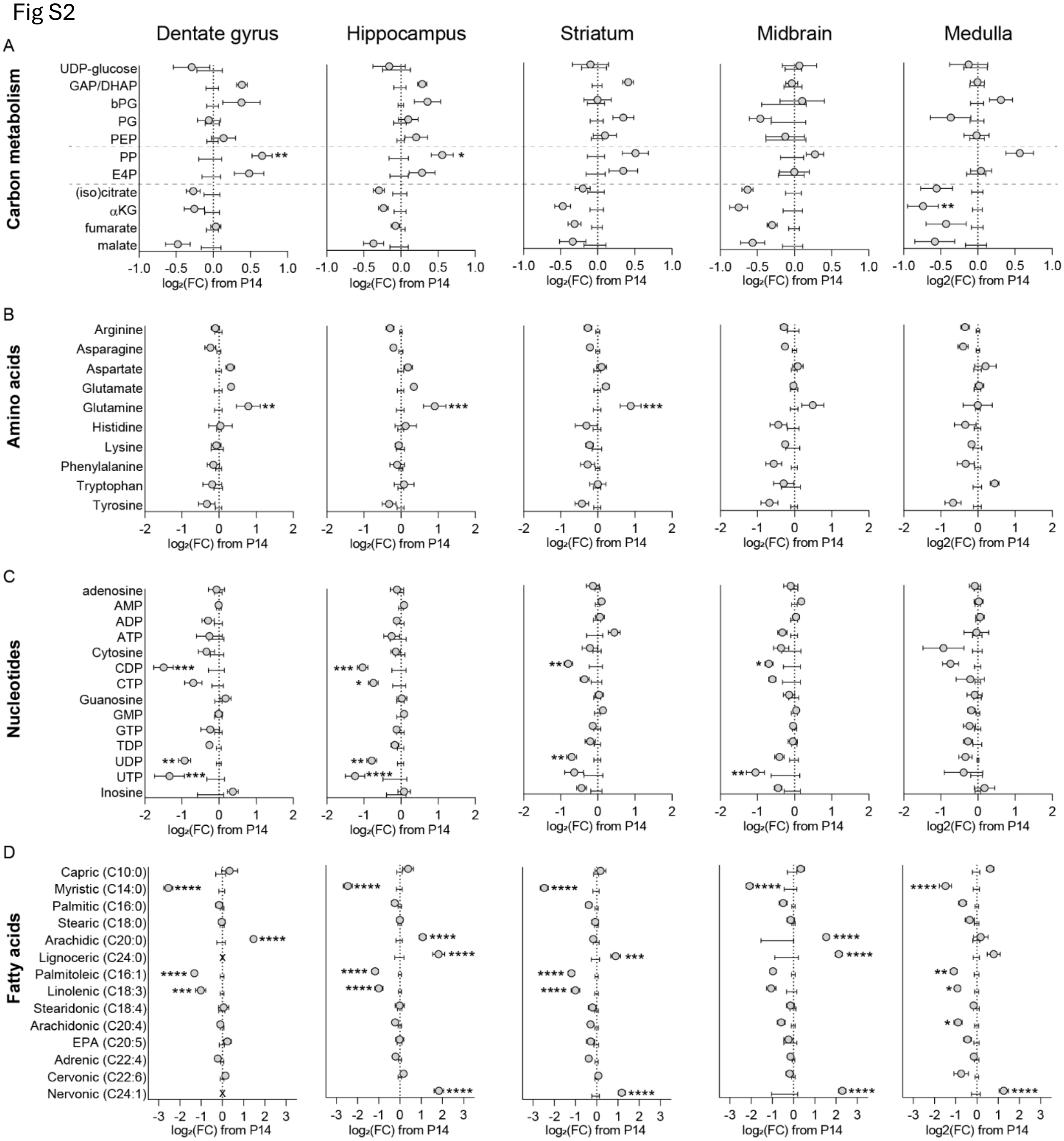

Figure S2 (associated with Fig 2): Targeted metabolomic analysis of the dentate gyrus, hippocampus, striatum, midbrain, and medulla. A-D) Data from P28 brains (grey circles) were plotted relative to P14 ion intensities. Analysis was performed for carbon metabolism (A), amino acids (B), nucleotides (C), and fatty acids (D). *p<0.05, **p<0.01, ***p<0.001, ****p<0.0001 by individual t-tests, with Holm-Šidák multiple comparison correction. N = 6 P14 (4 female, 2 male) and 6 P28 (4 female, 2 male). GAP/DHAP: glyceraldehyde-3-phosphate/dihydroxyacetone phosphate; bPG: bisphosphoglycerate; PG: phosphoglycerate; PEP: phosphoenolpyruvate; PP: pentose phosphates; α-KG: α-ketoglutarate.

Fig S3

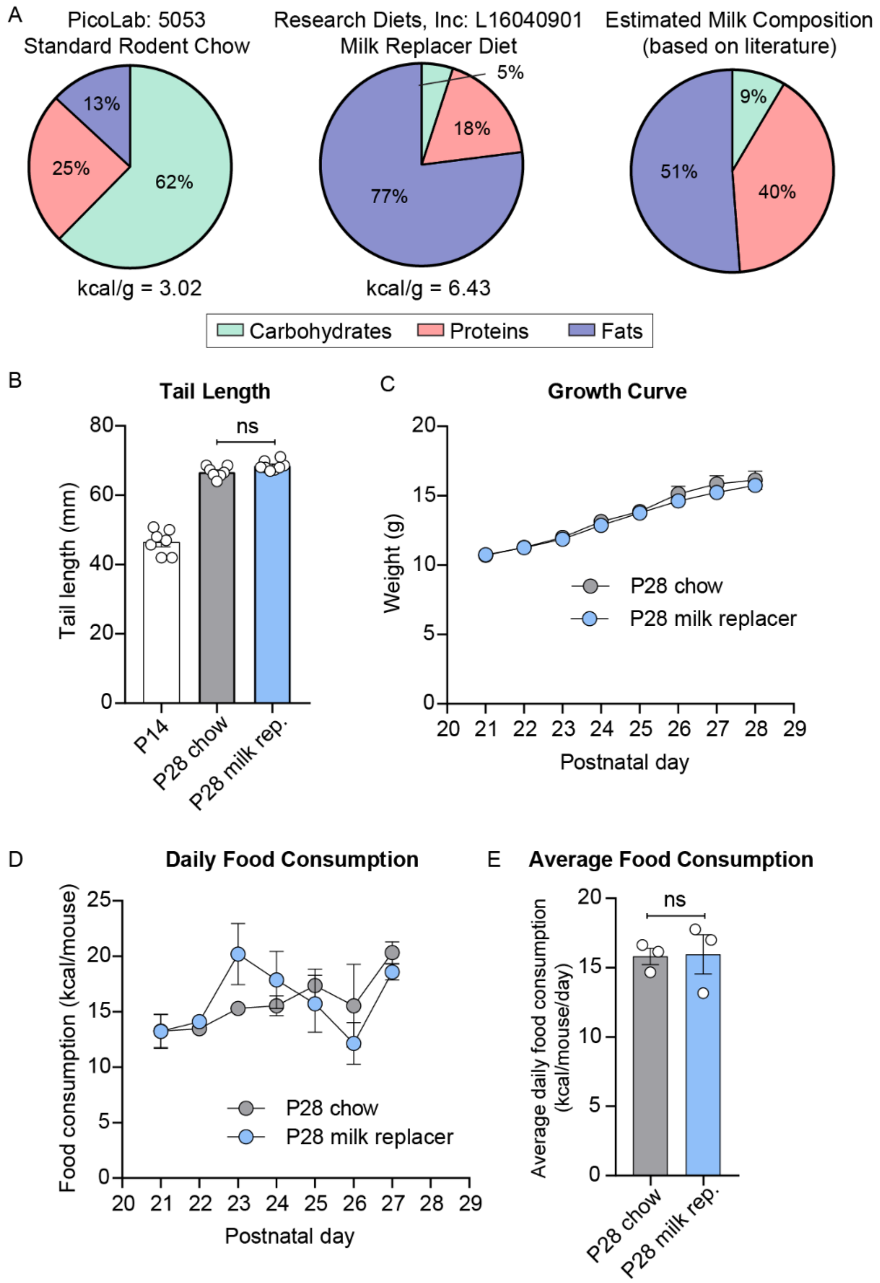

Figure S3 (associated with Fig 5): Comparison of diet composition, food intake, and growth. A) Macronutrient composition of standard rodent chow, milk replacer diet, and estimated composition of maternal milk. B) Tail length on collection day and C) body weight across developmental time points. D) Daily and (E) average food intake measured in kilocalories (measured for each cage and divided by number of mice housed in that cage). Data compared by two-way Student's t-test. N = 7 P14 (4 female, 3 male), 7 P28 chow (3 female, 4 male) and 9 P28 milk replacer diet (6 female, 3 male); conducted as 3 separate experiments.

Fig S4

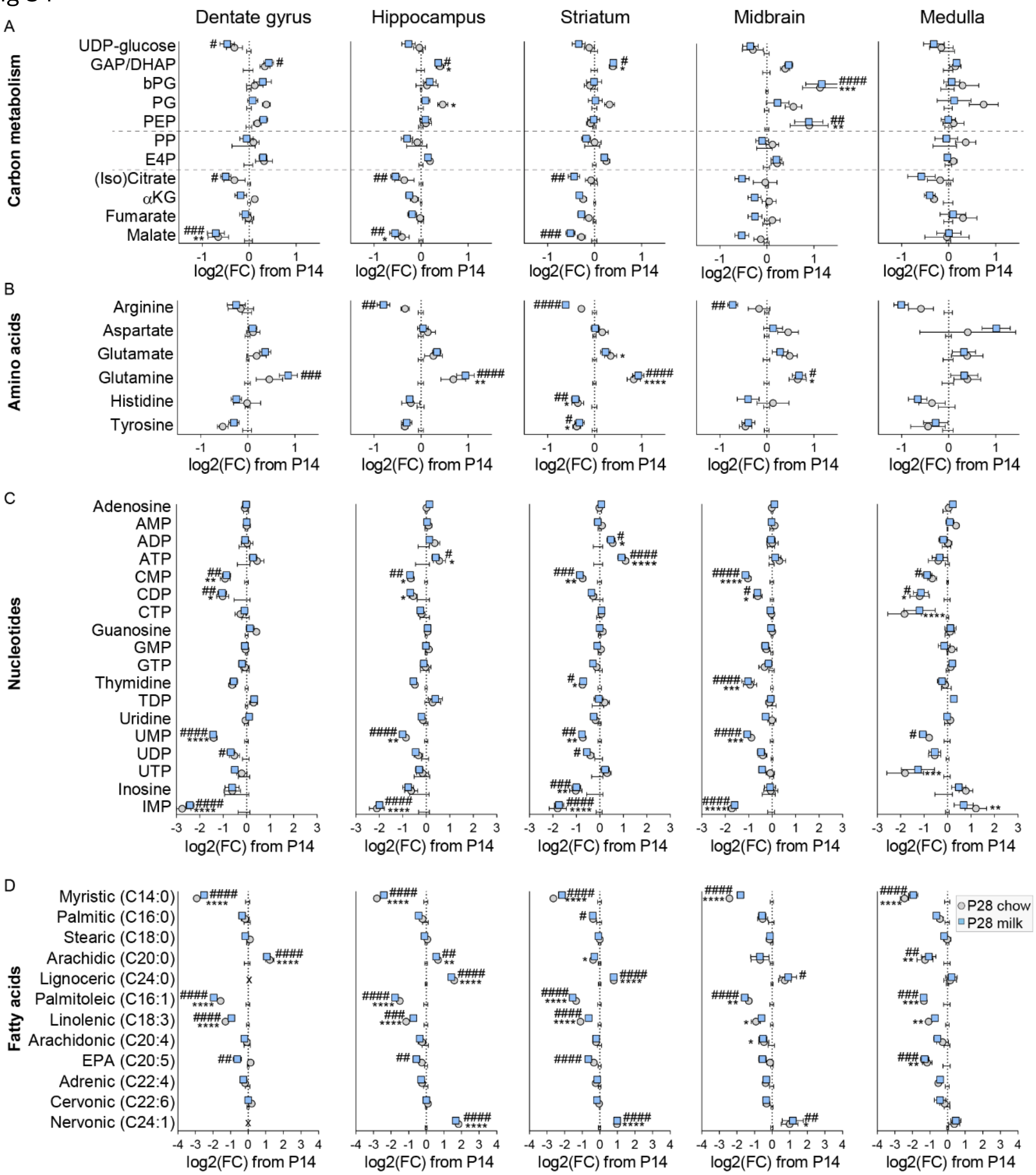

Figure S4 (associated with Fig 5): Brain metabolic profiles during postnatal development under standard chow and milk replacer diets. Targeted metabolomics of the dentate gyrus, hippocampus, striatum, midbrain, and medulla with P28 standard chow mice (grey circles) and P28 milk replacer mice (blue squares) plotted relative to P14. Analysis was performed for carbon metabolism (A), amino acids (B), nucleotides (C), and fatty acids (D). *p<0.05, **p<0.01, ***p<0.001, ****p<0.0001 by two-way ANOVA with Tukey multiple comparison correction. N = 7 P14 (4 female, 3 male), 7 P28 chow (3 female, 4 male) and 9 P28 milk replacer diet (6 female, 3 male); conducted as 3 separate experiments. GAP/DHAP: glyceraldehyde-3-

phosphate/dihydroxyacetone phosphate; bPG: bisphosphoglycerate; PG: phosphoglycerate; PEP: phosphoenolpyruvate; PP: pentose phosphates; α-KG: α-ketoglutarate.
